# RNA sequencing of isolated cell populations expressing human *APOL1* G2 risk variant reveals molecular correlates of sickle cell nephropathy in zebrafish podocytes

**DOI:** 10.1371/journal.pone.0217042

**Published:** 2019-06-03

**Authors:** Joseph L. Bundy, Blair R. Anderson, Ludmila Francescatto, Melanie E. Garrett, Karen L. Soldano, Marilyn J. Telen, Erica E. Davis, Allison E. Ashley-Koch

**Affiliations:** 1 Duke Molecular Physiology Institute, Duke University Medical Center, Durham, North Carolina, United States of America; 2 Department of Medicine, Duke University Medical Center, Durham, North Carolina, United States of America; 3 Center for Human Disease Modeling, Duke University Medical Center, Durham, North Carolina, United States of America; University Medicine Greifswald, GERMANY

## Abstract

Kidney failure occurs in 5–13% of individuals with sickle cell disease and is associated with early mortality. Two *APOL1* alleles (G1 and G2) have been identified as risk factors for sickle cell disease nephropathy. Both risk alleles are prevalent in individuals with recent African ancestry and have been associated with nephropathic complications in other diseases. Despite the association of G1 and G2 with kidney dysfunction, the mechanisms by which these variants contribute to increased risk remain poorly understood. Previous work in zebrafish models suggest that the G2 risk allele functions as a dominant negative, whereas the G1 allele is a functional null. To understand better the cellular pathology attributed to *APOL1* G2, we investigated the *in vivo* effects of the G2 risk variant on distinct cell types using RNA sequencing. We surveyed *APOL1* G2 associated transcriptomic alterations in podocytes and vascular endothelial cells isolated from zebrafish larvae expressing cell-type specific reporters. Our analysis identified many transcripts (n = 7,523) showing differential expression between *APOL1* G0 (human wild-type) and *APOL1* G2 exposed podocytes. Conversely, relatively few transcripts (n = 107) were differentially expressed when comparing *APOL1* G0 and *APOL1* G2 exposed endothelial cells. Pathway analysis of differentially expressed transcripts in podocytes showed enrichment for autophagy associated terms such as “Lysosome” and “Phagosome”, implicating these pathways in *APOL1* G2 associated kidney dysfunction. This work provides insight into the molecular pathology of *APOL1* G2 nephropathy which may offer new therapeutic strategies for multiple disease contexts such as sickle cell nephropathy.

## Introduction

Individuals of African descent are at greater risk for developing end-stage renal disease (ESRD) than individuals of European descent [1]. This disparity in risk has been associated, at least in part, with genetic variation in Apolipoprotein L1 (*APOL1*) [2]. *APOL1* is expressed in a variety of tissues, including the brain, liver, and kidney,[3] and contains a secretory domain that permits its release into circulation. Although there is relatively little known about APOL1 function, circulating APOL1 protein is a minor component of high density lipoprotein (HDL). In addition to its secretion into the bloodstream, *APOL1* is also expressed in both podocytes and endothelial cells in the kidney [4]. C-terminal mutations in APOL1 are associated with a constellation of complex diseases of the kidney, including chronic kidney disease (CKD), HIV-associated nephropathy (HIVAN), and focal segmental glomerulosclerosis (FSGS) [2, 5, 6].

There are two nephropathy-associated variants of *APOL1*, referred to as *APOL1* G1 and G2 [7]. *APOL1* G1 (G1) is comprised of two single nucleotide polymorphisms (SNPs) which are in linkage disequilibrium (LD) [6] and results in the amino acid substitutions S342G and I384M near the C-terminus. *APOL1* G2 (G2) is characterized by a six-nucleotide deletion resulting in the loss of two codons, N388 and Y389. Both the G1 and G2 variants are exclusive to individuals with recent African ancestry. G1 and G2 have allele frequencies of 23% and 13% in African Americans, respectively; and display complete negative LD (to date, they have not been observed on the same chromosome) [2, 6]. Thus, an individual has at most two copies of known *APOL1* risk variants (G1/G1, G1/G2, or G2/G2). Individuals with these genotypes have a 20-fold increased risk for developing FSGS or HIVAN relative to individuals with homozygous wild-type *APOL1* (G0) [6]. The maintenance of these alleles in African populations is attributable to the observation that both risk variants confer partial resistance to *T*. *b*. *rhodensiense*, a parasitic microorganism that causes African sleeping sickness. Consequently, G1 and G2 confers a selective advantage for individuals with these variants in endemic regions of Africa, despite their strong association with kidney disease [2].

The genetic association between *APOL1* and kidney dysfunction has been explored widely. However, the molecular mechanism by which these variants confer risk remains poorly understood. To shed light on *APOL1* function, we used the zebrafish animal model to explore the effects of *APOL1* risk variants and previously identified an *APOL1* orthologue in zebrafish [8]. We have demonstrated previously that morpholino (MO) mediated gene suppression of zebrafish *apol1* results in kidney associated phenotypes, such as transcriptional perturbation, edema and podocyte effacement [8]. Using this model, we tested whether co-injection of human *APOL1* mRNA encoding risk variants could significantly ameliorate edema induced by MO mediated gene suppression of endogenous zebrafish *apol1*. Neither G1 nor G2 *APOL1* mRNAs ameliorated MO induced edema, suggesting that both these variants result in *APOL1* loss of function. However, injection of *APOL1* G2 mRNA alone was sufficient to induce edema in larvae at 5 days post fertilization (dpf), even in the absence of anti-*apol1* MO. Thus, G2 appears to be a mechanistically distinct dominant negative variant compared to G1, which scored as functional null in this assay. This hypothesis is supported by independent investigations of *APOL1* associated kidney dysfunction in zebrafish. Olabisi and colleagues (2016) investigated pronephric microstructure in transgenic zebrafish expressing *APOL1* G0, G1, or G2, and reported distinct pathological features in each risk variant [9]. The hypothesis that G1 and G2 variants have distinct physiologic effects is also supported by other model systems. One investigation in podocyte cell lines overexpressing either G1 or G2 demonstrated lower cell viability with G2 relative to G1 [10]. This hypothesis is further supported by observational studies, finding that G0/G2 sickle cell patients are significantly more likely to develop nephropathy than their G0/G1 counterparts [11]. Together, these data suggest that the G1 and G2 risk variants are pathomechanistically distinct. Despite these findings, the practice of pooling G1 and G2 expressing individuals into a single high-risk *APOL1* group persists both in human genetic studies [12, 13] and in animal model based investigations [14].

It also unclear what population(s) of cells are principally affected by expression of *APOL1* risk variants. Because *APOL1* is expressed ubiquitously and secreted into systemic circulation, it is challenging to identify the populations of cells principally affected. Recent molecular investigations of *APOL1* suggest that podocytes and endothelial cells of the kidney are likely affected cell types [14]. Retrospective transplantation studies have shown that *APOL1* associated risk for nephropathy is conferred by the genotype of donor tissue,[15] suggesting that nephropathy is likely induced by locally translated APOL1 within populations of kidney cells [16]. To better understand the role of G2 associated kidney dysfunction in these cell types, we used fluorescence activated cell sorting (FACS) coupled with RNA sequencing (RNA-seq) to explore transcriptomic perturbations in distinct kidney cell types in G2 mRNA injected zebrafish larvae.

## Results

**Fig 1:**
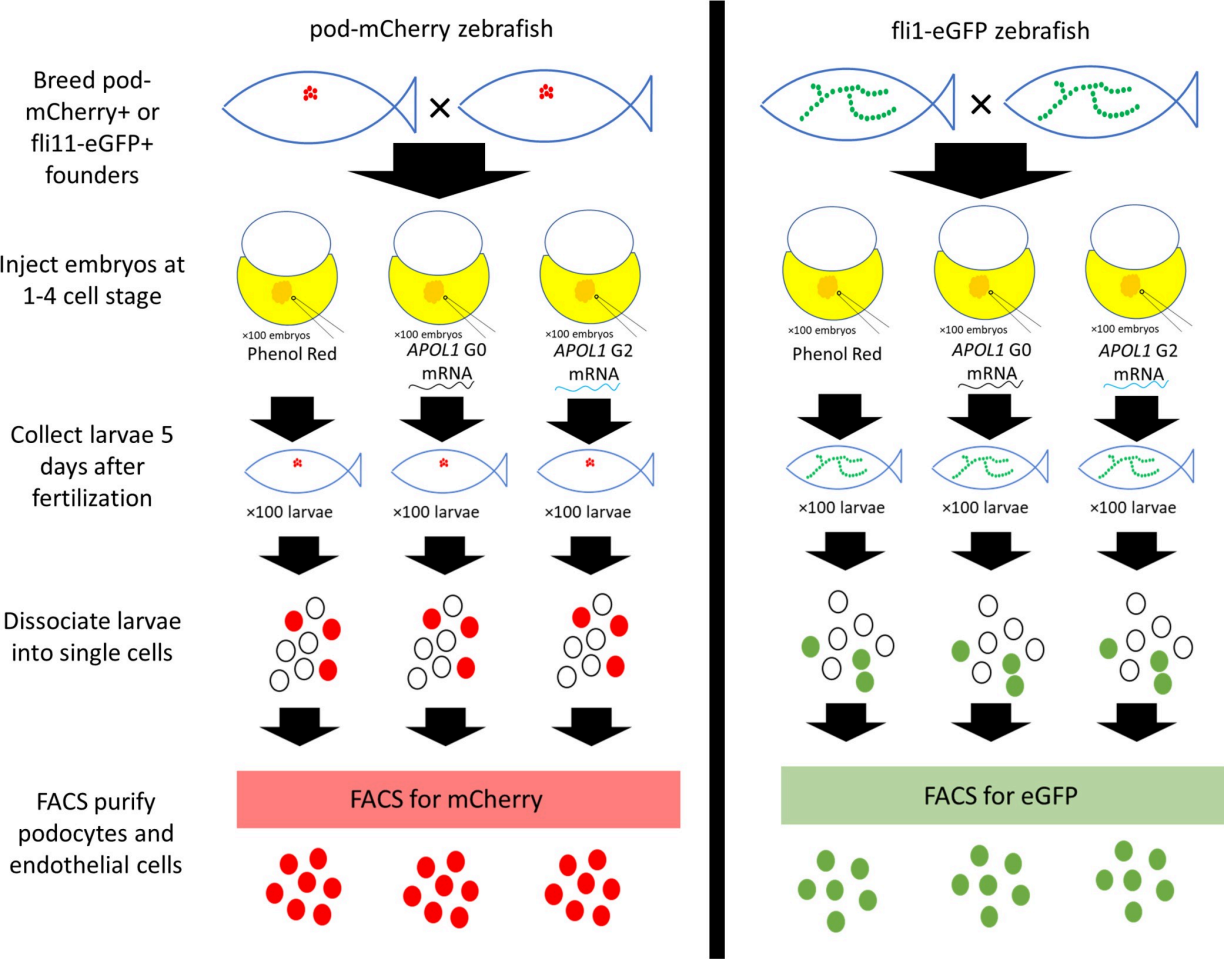
Schematic of sample preparation and experimental design. Zebrafish expressing either pod-mCherry or fli1-eGFP were injected with *APOL1* mRNA and subsequently dissociated at 4 days after fertilization. Dissociated cells were FACS based on mCherry and eGFP expression into samples of podocytes and endothelial cells, respectively.

**Fig 2:**
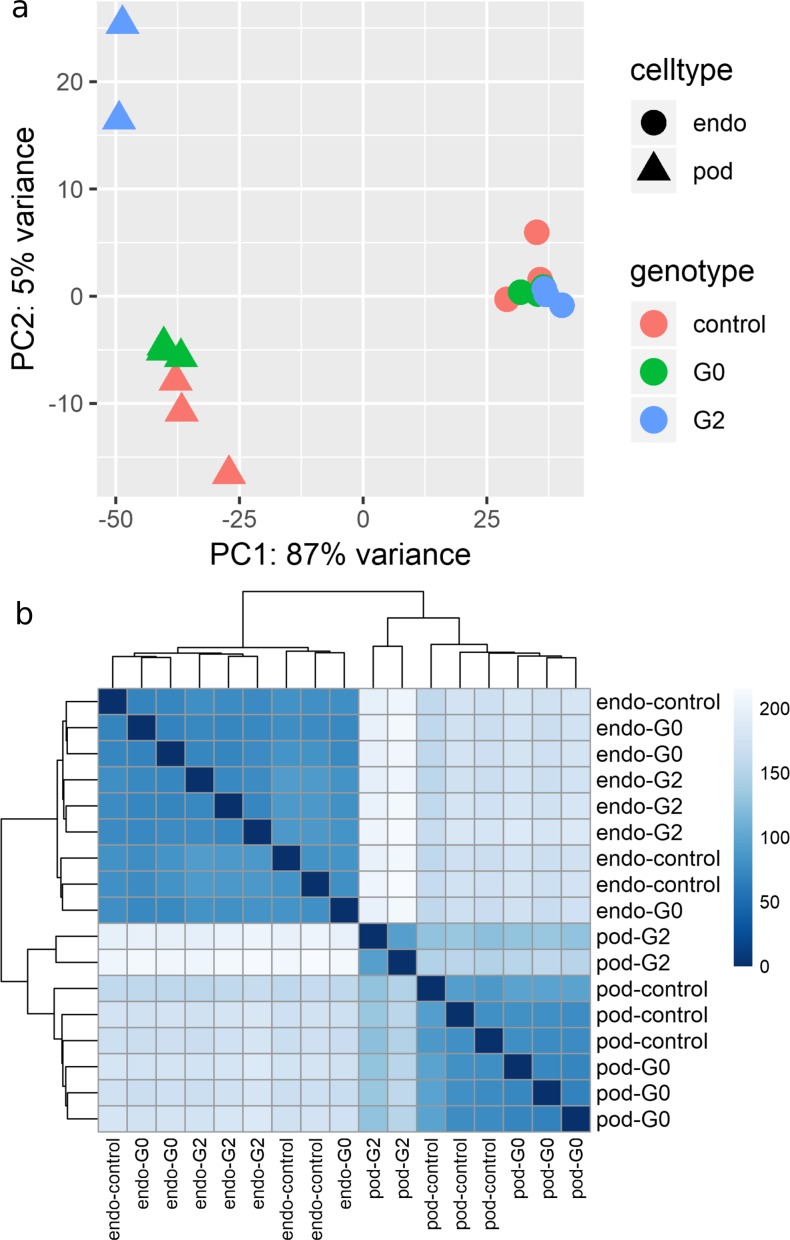
Clustering analysis of cDNA libraries. a) Principal component analysis (PCA) of cDNA libraries. PCA was based on the top 500 most variant transcripts in the dataset. Cell type is encoded by shape. Endothelial cells are represented by circles and podocytes are represented by triangles. Injection treatment is encoded by color. Phenol red injected (control) samples are colored red. Samples injected with G0 and G2 are colored green and blue, respectively. b) Sample correlation and hierarchical clustering analysis.

We conducted transcriptomic analysis of FACS purified podocytes and vascular endothelial cells from 4 dpf zebrafish larvae exposed at the 1–4 cell stage to *APOL1* G0 mRNA, *APOL* G2 mRNA, or phenol red (n = 3 pools of 100 larvae per cell type, per condition, Fig 1). All 18 resulting cDNA libraries were sequenced on an Illumina HiSeq 2500 yielding a total of 316,464,722 reads across all samples. Reads uniquely aligned to 27,615 zebrafish transcripts with at least one aligned read count. Independent alignment of sequenced reads against human *APOL1* mRNA revealed no persistent human mRNA in injected samples, suggesting that the injected human mRNA is lost by 4 dpf. Despite standardizing the amount of cDNA loaded for every sample, sequencing depth was variable across samples (S1 Fig). One G2 podocyte sample was excluded due to low read counts and a low percentage of unique reads. To visualize global differences in gene expression profiles, we conducted a principal component analysis (PCA) of the 500 genes with the greatest variance in the dataset (Fig 2A). As expected, libraries prepared from FACS purified podocytes and endothelial cells clustered distinctly along principal component 1, which explained 89% of the variance in the dataset. Of note, all libraries prepared from endothelial cells clustered together regardless of mRNA treatment. However, libraries prepared from podocytes treated with G2 mRNA clustered distinctly from control and G0 exposed podocytes along principal component 2. This observation was replicated by an unsupervised clustering analysis (Fig 2B). Taken together, these data suggest that G2 mRNA alters the transcriptomic signature more strongly in podocytes than in endothelial cells.

To identify transcripts with altered expression in the presence of human *APOL1* mRNA, we conducted pairwise comparisons between treatment groups within each cell type. A recent investigation of *APOL1* expression in HEK293 cells demonstrated that *APOL1* G0 can induce cytotoxicity if over expressed [17]. Therefore, we compared the sham injected samples with G0 mRNA injected samples to ascertain if G0 induces similar deleterious effects in zebrafish. We identified 199 and 155 differentially expressed (DE) transcripts in endothelial cells and podocytes, respectively. We contrasted these lists of DE transcripts to determine if the same transcripts were altered by G0 injection in each cell type. Only one transcript (*nfil3-6*) was found in common between both lists of DE transcripts, constituting little concordance between cell types. To identify biological processes that were overrepresented in the sets of DE transcripts, we conducted gene ontology enrichment and pathway analyses (GOEA) on each set. No significant enrichment was detected for either cell type. The set analysis combined with the lack of significant enrichment of gene ontology categories in the lists of DE transcripts suggests that G0 mRNA has a relatively weak, if not spurious, biological effect on the zebrafish transcriptome.

To identify transcriptomic perturbations induced by expressing the G2 risk variant, we compared G0 and G2 injected samples in each cell type. In endothelial cells, 107 transcripts showed significantly perturbed expression. The majority of these transcripts (79%) were down-regulated in G2 injected samples relative to G0. GOEA analysis returned no significant enrichment of distinct biological processes, suggesting relatively minor and nonspecific perturbation of molecular networks by G2 in endothelial cells.

**Fig 3:**
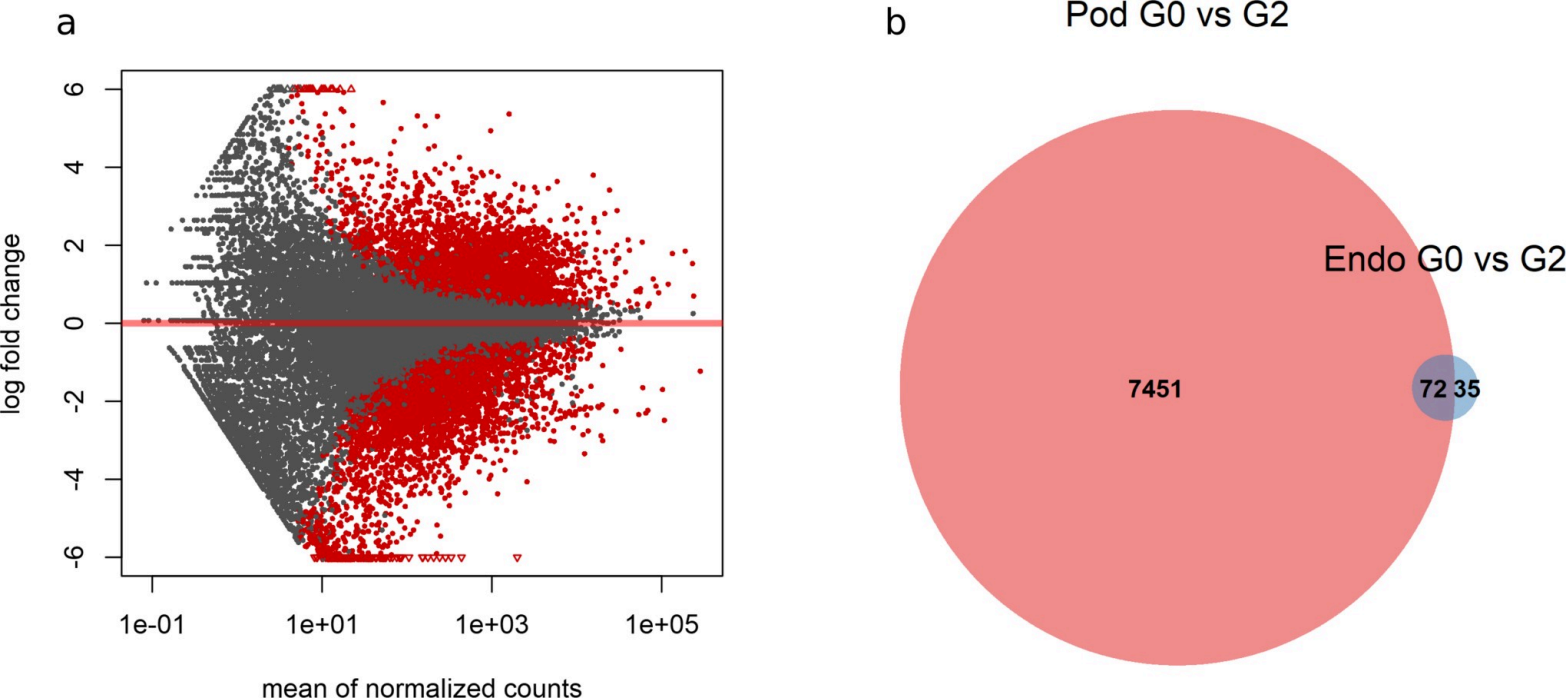
Comparison of G0 and G2 exposed cells. a) Shotgun plot of transcript expression. Points colored red are transcripts that are significantly upregulated (above y = 0) or downregulated (below y = 0) at a FDR-adjusted p-value of less than 0.05. b) Venn diagram comparing genes perturbed by G2 expression in endothelial cells and podocytes.

In contrast to the modest changes observed in endothelial cells, podocytes exposed to G2 showed evidence of substantial transcriptional alteration. Comparison of G0 and G2 exposed podocytes identified 7523 differentially expressed transcripts (Fig 3A, red dots). Of these transcripts, approximately the same number were found to be up-regulated (Fig 3A, red points above y = 0, n = 3943) as down-regulated (Fig 3A, red points below y = 0, n = 3580) in G2 exposed podocytes. To determine the extent to which the relatively few transcripts that were perturbed in G2 exposed endothelial cells were also perturbed in podocytes, we conducted a set analysis of the two transcript lists. Of the 107 transcripts significantly perturbed in endothelial cells, two-thirds (72 of 107) also showed perturbed expression in podocytes (Fig 3B). This suggests that while podocytes are principally affected by G2 expression, a subset of transcripts we observed to be perturbed in podocytes may represent a more ubiquitous response to G2.

Among the top DE transcripts as ranked by q-value, is nephrin (*nphs1*), as well as other genes previously associated with kidney disease (Table 1). Nephrin is known to be associated with kidney dysfunction [18, 19], and has been shown to be down-regulated in the podocytes of *apol1* knockdown larvae, as well as in kidneys of patients with CKD [20]. Surprisingly, nephrin is up-regulated in G2 injected larvae relative to G0 in our dataset. Additionally, we found endogenous *apol1* to be among additional transcripts perturbed significantly by exposure to G2, which shows significant elevation in G2 exposed podocytes relative to G0 (but in no other comparison). This constitutes a possible mechanism of compensation for G2, which our previous investigations suggest has dominant-negative function [8]. Furthermore, several modulators of the adaptive immune system, such as *il6r* and *il4*, were differentially expressed; transcripts have been associated previously with the development of CKD [21–23].

**Table 1:** Transcripts perturbed significantly by G2 expression in zebrafish podocytes.

Gene ID	Gene symbol	Gene name	q value	Log2 Fold Change
ENSDARG00000036940	ctss1	cathepsin S, ortholog 1	2.96E-42	3.15
ENSDARG00000078525	noct	nocturnin	2.30E-38	3.19
ENSDARG00000053542	kctd12.2	potassium channel tetramerisation domain containing 12.2	3.11E-35	2.27
ENSDARG00000101754	BX664622.3	NA	3.14E-33	3.02
ENSDARG00000093124	scpp8	secretory calcium-binding phosphoprotein 8	3.16E-33	3.37
ENSDARG00000060758	nphs1	nephrosis 1, congenital, Finnish type (nephrin)	1.42E-31	4.60
ENSDARG00000104474	il6r	interleukin 6 receptor	1.42E-31	3.34
ENSDARG00000087909	il4	interleukin 4	1.67E-31	3.28
ENSDARG00000073978	crabp2a	cellular retinoic acid binding protein 2, a	8.64E-31	-3.18
ENSDARG00000075748	nckap1l	NCK associated protein 1 like	1.08E-30	2.30

GOEA of the full set of differentially expressed transcripts (regardless of direction of change) revealed a large number of significantly enriched ontologies (Table 2). These ontologies included molecular functions such as “Calcium ion binding”, and “ion transmembrane transporter activity.” Among the transcripts associated with “ion transmembrane transporter activity” were sodium, potassium, calcium, and chloride ion channels. Disruption of ionic gradients has been associated previously with overexpression of *APOL1* in HEK293 cells, suggesting the presence of a conserved mechanism of molecular pathology. In addition to GOEA, we considered that our differentially expressed transcripts might be enriched for distinct biological pathways, which we also explored using WebGestalt. This analysis yielded a functionally diverse set of significantly enriched pathways, such as “Metabolic pathways” (p = 5.42e-08), “Lysosome” (p = 6.08e-06), and “Phagosome” (p = 6.38e-06),” (Table 2).

**Table 2:** Significantly enriched gene ontology and KEGG pathway terms.

Ontology/Pathway	ID	FDR q-value	#Genes	Enrichment Score
Extracellular region part	GO:0044421	2.2e-08	173	1.45
Metabolic pathways	dre01100	5.42e-08	541	1.20
Synapse	GO:0045202	5.02e-07	101	1.57
Neuron part	GO:0097458	1.72e-06	106	1.52
Calcium ion binding	GO:0005509	1.47e-06	174	1.40
Molecular function regulator	GO:0098772	1.47e-06	228	1.33
Lysosome	dre04142	6.08e-06	76	1.61
Carbon metabolism	dre01200	6.08e-06	73	1.63
Phagosome	dre04145	6.38e-06	77	1.60
Endoplasmic reticulum	GO:0005783	2.3e-04	168	1.29
Plasma membrane region	GO:0098590	5.29e-04	63	1.51
Chemorepellent activity	GO:0045499	1.36e-03	16	2.29
Ion transmembrane	GO:0015075	1.7e-03	206	1.23

## Discussion

The current study describes transcriptional perturbation of zebrafish larvae exposed to human *APOL1* mRNA. Previous investigations have shown that *APOL1* overexpression significantly reduced cell viability in HEK293 cells, irrespective of the presence of risk variants [17]. In this context, we were surprised to find relatively few transcripts with perturbed expression as a result of exposure to G0 in either cell type. However, there are numerous differences in experimental design between O’Toole et al. (2018) and the current study (model system, mechanism of *APOL1* expression) that may explain the lack of more substantial transcriptional perturbation in our data [17].

**Fig 4:**
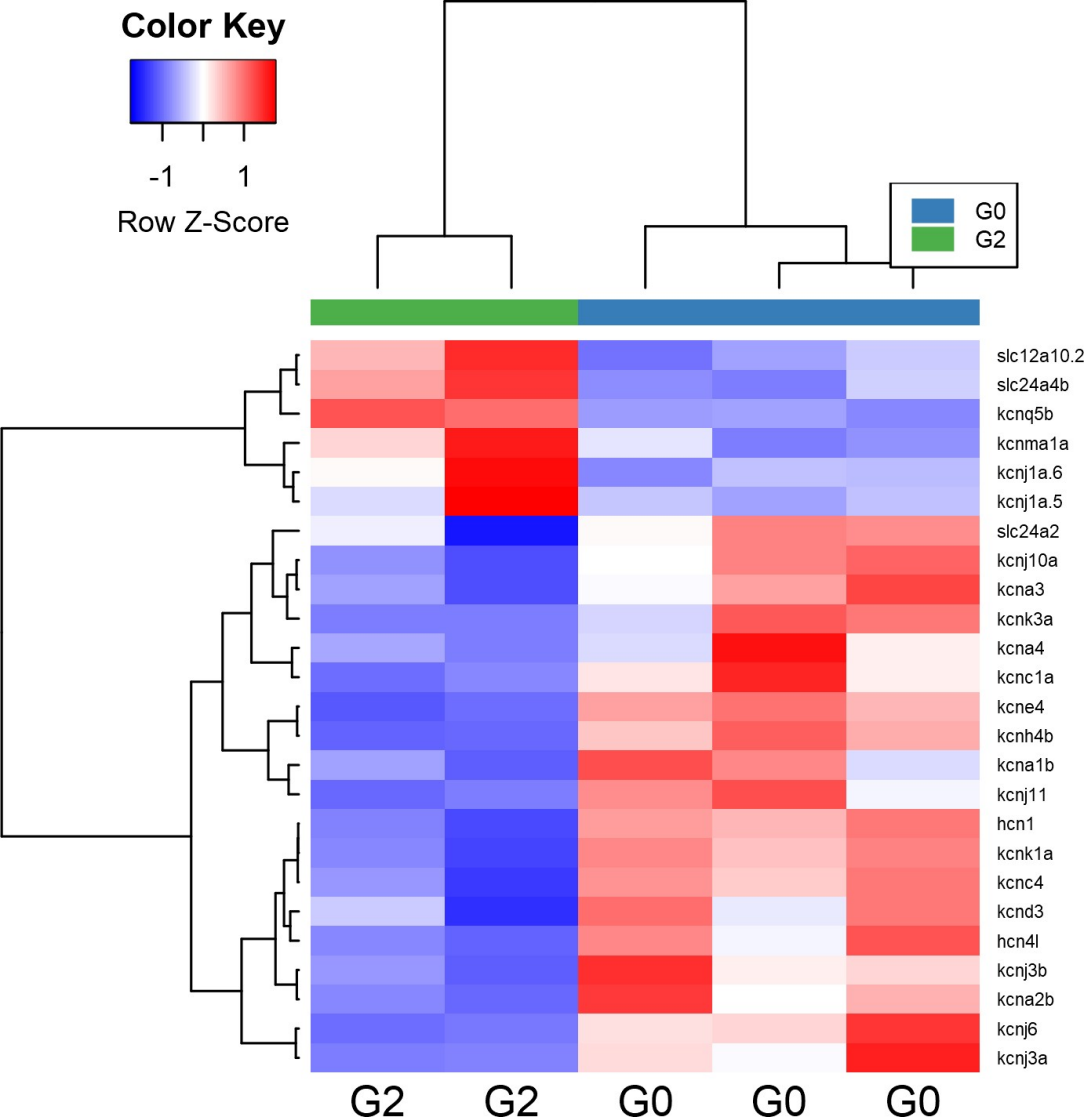
Heatmap for transcriptionally perturbed K+ channels. Samples are organized into columns, and transcripts are grouped by rows. Boxes are shaded by log2 fold change from row-centered mean. The majority (n = 19 of 25) transcripts are significantly down-regulated in podocytes exposed to G2.

In contrast to the relatively mild transcriptional alterations induced by G0, exposure to the G2 risk variant showed widespread changes in mRNA levels in podocytes but not endothelial cells in 5 dpf larvae. Among the over 7,500 transcripts showing perturbation in response to G2, an overrepresented subset of these transcripts are associated with the regulation of ion channels. APOL1 protein is known to form ion permeable pores in endosomal membranes of trypanosomes, allowing endosomal contents to leak into the cytoplasm and disrupt the molecular milieu [24]. Pore formation has also been reported to occur in HEK293 cells when *APOL1* is over-expressed,[17] bolstering an emerging hypothesis that *APOL1*-associated kidney damage may be due to toxic *APOL1*-mediated pore formation [25]. However, previous investigations have yielded conflicting results with regard to risk-variant mediated disruption of ion concentrations. Some studies have shown that expression of *APOL1* G1 and G2 results in the loss of intracellular K^+^ ions relative to G0 expressing controls,[10] while other studies failed to find significant differences in K^+^ concentration between G0 and G1/G2 expressing cells [17]. Although the current study does not measure intracellular K^+^ concentrations directly, our data support the hypothesis that G2 affects the regulation of intracellular K^+^. Twenty-five K^+^ channels were found to have significantly perturbed expression in G2 exposed podocytes, the majority (76%) of which are down regulated (Fig 4A). It is known that one molecular mechanism for maintaining ionic homeostasis is the regulation of ion channel expression [26]. In this context, the observed down-regulation of K^+^ channels may be a compensatory mechanism by which zebrafish podocytes selectively reduce K^+^ membrane permeability in response to *APOL1* G2 mediated loss of intracellular K^+^.

Among the significantly enriched gene ontology terms were “lysosome” and “phagosome”. Among the lysosome-associated genes are 11 cathepsin isoforms, all of which were up-regulated in G2 exposed samples. Cathepsins are known to be critical for lysosomal processing of protein. A previous study of *APOL1* expressing transgenic mice found that podocytes expressing *APOL1* risk variants have deficits in autophagic flux, and that risk-variant containing *APOL1* showed preferential localization to late endosomes [14]. Furthermore, this same mouse study showed that turning off *APOL1* expression in transgenic mice could reverse phenotypes indicative of kidney dysfunction, suggesting that *APOL1* mediated kidney dysfunction requires persistent APOL1 expression and is not derived from lasting developmental perturbations [14]. This hypothesis is consistent with a study conducted by Kotb et al (2016), in which knockdown of zebrafish mRNA at 3 dpf was shown to induce edema within 3 hours [20]. In contrast with these experimental paradigms, our experiments involved a one-time injection and over-expression of *APOL1* mRNA immediately after fertilization, followed by cellular dissociation and RNA extraction four days later. An independent alignment of sequenced reads from our *APOL1* mRNA-injected zebrafish against the human *APOL1* sequence revealed no detectable human *APOL1* mRNA in any cDNA libraries, suggesting that *APOL1* translation was unlikely to be occurring at the time of library preparation. However, we have previously shown that microinjected *APOL1* mRNA at the 1–4 cell stage is translated and detectable 2 days post fertilization [8]. Nevertheless, our data indicate substantial transcriptional differences between G0 and G2 exposed samples even in the absence of *APOL1* mRNA at the time of our experimental endpoint. These observations suggest that at least some effects of G2 exposure may persist even in the absence of *APOL1* expression.

Our RNA-seq based investigation of G2 associated molecular pathology provides a broad view into patterns of dysregulated gene expression in the zebrafish kidney, identifying over 7000 differentially expressed transcripts. However, our study is not without limitations. To isolate endothelial cells, we used a zebrafish line expressing eGFP under the control of the *fli1* promoter, which is not expressed exclusively in glomerular endothelial cells. Thus, our endothelial RNA-seq samples are derived from both glomerular endothelia as well as endothelia located outside the kidney. Additionally, we attempted to validate a subset of transcripts that were found to be differentially abundant between G1 and G0 injected podocytes with quantitative reverse transcription polymerase chain reaction (qRT-PCR). Extracting sufficient mRNA from FACS purified podocytes was challenging, as only a small percent (~0.5%) of cells prior to sorting were positive for the podocyte specific reporter (mCherry). Without amplification of cDNA prior to qRT-PCR analysis, there was insufficient material to confirm the differential expression we observed in the RNA-seq experiment. Additionally, the current experiment focuses only on the molecular changes associated with exposure to G2. In the context of our previous findings that suggest G1 and G2 have distinct pathomechanisms, additional investigations focusing on molecular changes associated with each variant would help to understand the molecular effects of these variants.

In aggregate, our data confirm and supplement the findings of other independent investigations. Our data demonstrate that zebrafish kidney cells expressing *APOL1* G2 in the developing zebrafish show a significantly altered transcriptomic landscape relative to G0 counterparts. Measured by the number of transcripts showing altered expression, podocytes undergo extensive transcriptional perturbation compared to endothelial cells and appear to be the most likely cellular location of pathology in APOL1 risk-variant associated kidney disease.

## Methods

### *APOL1* messenger RNA synthesis

Production of human *APOL1* mRNA has been described previously [8]. Briefly, the *APOL1* G2 allelic construct was synthesized from an *APOL1* G0 human ORF clone (GenBank BC112943) using site-directed mutagenesis (QuikChange II, Stratagene), and subsequently transcribed (mMESSAGE mMACHINE, Life Technologies, Ambion) into capped mRNA.

### Zebrafish injections

This study was approved by the Duke University Institutional Animal Care and Use Committee protocol# A208-16-09. Two zebrafish lines expressing cell-type specific reporters enabled the isolation of specific cell populations by FACS (Fig 1). Podocytes and endothelial cells were isolated from dissociated zebrafish larvae expressing pod:NTR-mCherry [27] and TG(fli1:EGFP) [28], respectively. Embryos generated from natural mating of adult fish carrying each transgene received one of three experimental treatments by microinjection into the yolk: 1) human *APOL1* G0 mRNA, 2) human *APOL1* G2 mRNA, 3) phenol red (control). Injections were performed immediately after embryo collection during or prior to the 4 cell stage. All injections were performed with a WPI pneumatic pico pump microinjector calibrated to deliver 1 nL of *APOL1* G0 (150pg/nL) or 1 nL of *APOL1* G2 (150pg/nL) mRNA. After injection, embryos were incubated for 4 days at 28°C.

### Fluorescence-activated cell sorting (FACS)

Four dpf larvae were pooled (~100 larvae per sample) and incubated in 1.5mL tubes with deyolking buffer (55mM NaCl, 1.8mM KCl, 1.25mM NaHCO_3_) at 42°C for 5 minutes with gentle, periodic agitation. Deyolked larvae were then incubated for one hour with 1mL of dissociation buffer (0.25% trypsin in EDTA) at 42°C and agitated every 5 minutes by pipetting. Dissociated cells were put on ice and then centrifuged for 5 minutes at 660 x g at 4°C. The resulting pellet was resuspended in 1mL pre-chilled PBS + 5% heat-inactivated FCS. After resuspension, samples were centrifuged for 7 minutes at 200 x g at 4°C. Pellets were once again resuspended in 700uL PBS + 2% FCS, and subsequently pipetted through 40μm filter units (Falcon ref# 352340) to remove debris and non-dissociated material. Filtrate was concentrated by centrifugation for 7 minutes at 150 x g at 4°C. The resulting pellet was resuspended in 1mL PBS + 2% FCS and transferred to 1mL PBS + 2% FCS. Finally, all samples were spiked with 5uL of DNase1 (2mg/mL). For exclusion of non-viable cells during FACS, fli1-eGFP and pod-mCherry samples were spiked with 5uL 7AAD (1mg/mL) and 1uL of DAPI (5mg/mL), respectively. Isolation of both podocytes and endothelial cells was performed with a Beckman Coulter Astrios at the Duke Cancer Institute Shared Resource Flow Cytometry facility. Isolation of mCherry positive podocytes and eGFP positive endothelial cells used an excitation wavelength of 561nm and 488nm, respectively. Samples were sorted into 400uL of buffer RLT (Qiagen, ref# 74004), spiked with 5uL of β-mercapto-ethanol, and stored at -80°C until RNA-extraction.

### Library preparation

RNA was extracted from FACS purified samples using a Qiagen RNeasy micro kit (Qiagen, ref# 74004) and submitted to the Duke Center for Genomic and Computational Biology for library preparation and sequencing. cDNA libraries were prepared from mRNA using the SMARTSeq v.4 Ultra Low Input RNA Kit (Clonetech, cat# 634889) and sequenced on an Illumina HighSeq 2500.

### Data analysis

Quality control analysis of each sequenced library was performed using fastQC (FastQC, RRID:OMICS_01043; http://www.bioinformatics.babraham.ac.uk/projects/fastqc/). Removal of primer adapters was performed with Trim Galore (https://www.bioinformatics.babraham.ac.uk/projects/trim_galore/). Trimmed sequencing reads were aligned and mapped to the *Danio rerio* reference genome (release GRCm38) using the STAR alignment tool [29]. A separate alignment was performed against the human *APOL1* mRNA sequence. Following mapping with STAR, reads were filtered, sorted and indexed with Samtools (SAMTOOLS, RRID:nlx_154607; http://www.htslib.org/) [30]. Only reads that mapped to a single gene were utilized for further analysis. Uniquely mapped reads were used to generate counts for each annotated gene using HTSeq [31]. PCA and hierarchical clustering analyses were performed using DESeq2 [32]. The RNA-seq data supporting the conclusions of this article are available in the NCBI Gene Expression Omnibus [33] accession #GSE118000.

To identify transcripts that were differentially expressed between treatment groups, we performed pairwise comparisons with DESeq2 in the R statistical framework [32]. For all differential expression analyses, the criterion for considering a transcript to be differentially expressed was an FDR-adjusted p-value (q value) of less than 0.05.

### Gene ontology enrichment analysis

For Gene Ontology (GO) and KEGG (Kyoto Encyclopedia of Genes and Genomes Expression Database, RRID:nif-0000-21234) enrichment analyses, Ensembl gene identifiers for lists of differentially expressed genes were uploaded to WebGEstalt (WebGestalt: WEB-based GEne SeT AnaLysis Toolkit, RRID:nif-0000-30622; http://bioinfo.vanderbilt.edu/webgestalt/) [16, 34]. For both the GO and KEGG pathway enrichment analyses, the following criteria were used for filtering results: minimum number of genes per term = 5, p.adjust < 0.05. All transcripts detected with at least one read count in each pairwise comparison were used as the “background” list for GO and KEGG enrichment.

## Supporting information

S1 Fig Sequencing depth of cDNA libraries before and after normalization. (PNG)

S1 File ARRIVE guidelines checklist. (PDF)
