# Supplementary figures and images for: RNA sequencing of isolated cell populations expressing human APOL1 G2 risk variant reveals molecular correlates of sickle cell nephropathy in zebrafish podocytes
Source: PLoS One. 2019 Jun 3;14(6):e0217042. doi: 10.1371/journal.pone.0217042 (PMC6546218; doi:10.1371/journal.pone.0217042)

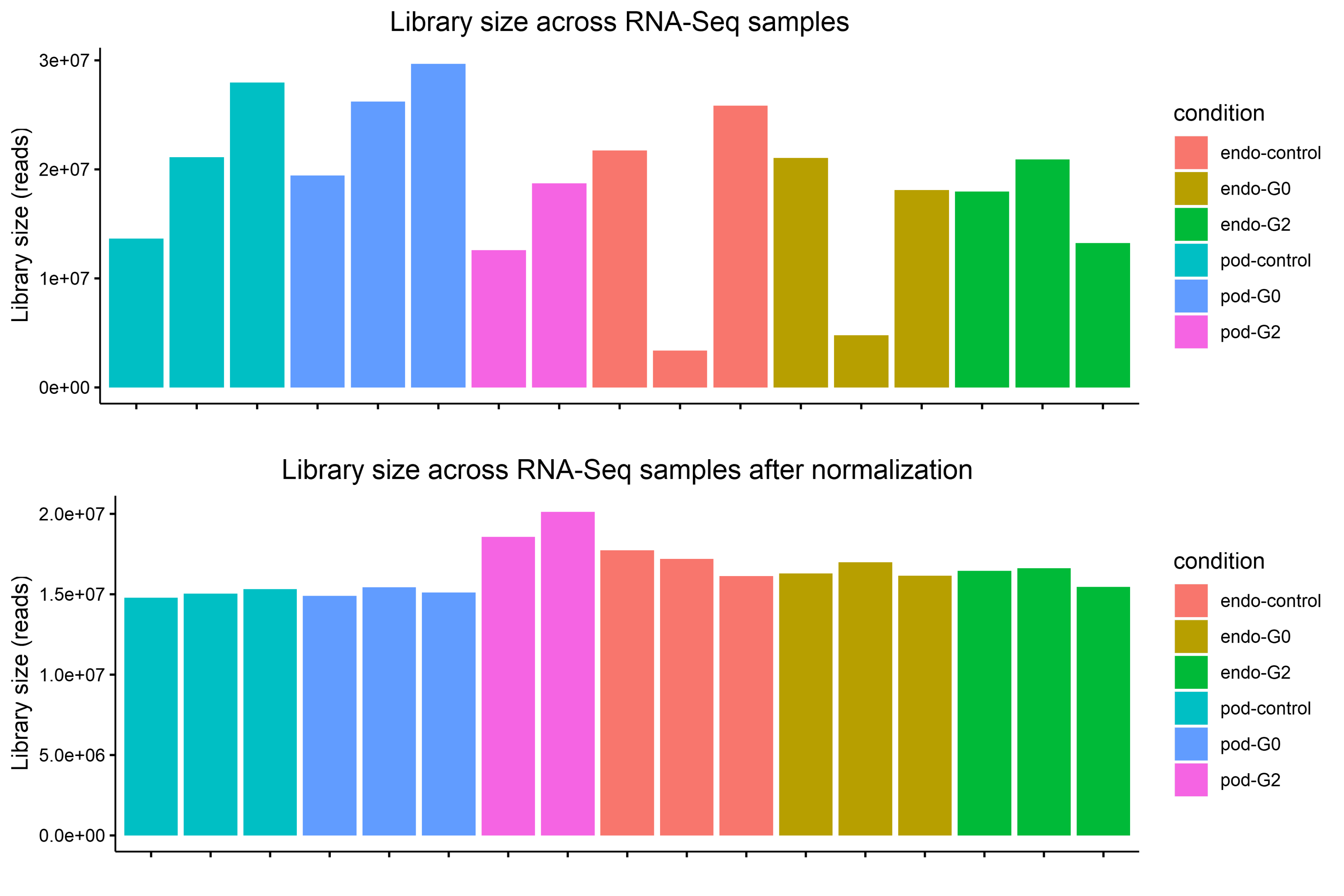

Supplement: S1 Fig — (PNG) [file pone.0217042.s001.png]
